# The less conserved metal-binding site in human CRISP1 remains sensitive to zinc ions to permit protein oligomerization

**DOI:** 10.1038/s41598-021-84926-y

**Published:** 2021-03-09

**Authors:** Jie Sheng, Bart M. Gadella, Nick K. Olrichs, Dora V. Kaloyanova, J. Bernd Helms

**Affiliations:** Department of Biomolecular Health Sciences, Faculty of Veterinary Medicine, Utrecht University, Utrecht, The Netherlands

**Keywords:** Protein aggregation, Biochemistry

## Abstract

Cysteine-rich secretory proteins (CRISPs) are a subgroup of the CRISP, antigen 5 and PR-1 (CAP) superfamily that is characterized by the presence of a conserved CAP domain. Two conserved histidines in the CAP domain are proposed to function as a Zn^2+^-binding site with unknown function. Human CRISP1 is, however, one of the few family members that lack one of these characteristic histidine residues. The Zn^2+^-dependent oligomerization properties of human CRISP1 were investigated using a maltose-binding protein (MBP)-tagging approach in combination with low expression levels in XL-1 Blue bacteria. Moderate yields of soluble recombinant MBP-tagged human CRISP1 (MBP-CRISP1) and the MBP-tagged CAP domain of CRISP1 (MBP-CRISP1^ΔC^) were obtained. Zn^2+^ specifically induced oligomerization of both MBP-CRISP1 and MBP-CRISP1^ΔC^ in vitro. The conserved His142 in the CAP domain was essential for this Zn^2+^ dependent oligomerization process, confirming a role of the CAP metal-binding site in the interaction with Zn^2+^. Furthermore, MBP-CRISP1 and MBP-CRISP1^ΔC^ oligomers dissociated into monomers upon Zn^2+^ removal by EDTA. Condensation of proteins is characteristic for maturing sperm in the epididymis and this process was previously found to be Zn^2+^-dependent. The Zn^2+^-induced oligomerization of human recombinant CRISP1 may shed novel insights into the formation of functional protein complexes involved in mammalian fertilization.

## Introduction

In mammals, before sperm become fully competent to fertilize an egg, spermatozoa must undergo a series of morphological, biochemical and physical changes in male and female tracts, known as epididymal maturation and capacitation, respectively^1–3^. During epididymal maturation, spermatozoa pass through the epididymis where an extensive remodeling of the sperm plasma membrane takes place, including the acquisition of epididymal proteins^4,5^. Some of these proteins contribute to the acquisition of sperm’s ability to bind and penetrate through the extracellular structures surrounding the oocyte, while others are involved in preventing the occurrence of premature capacitation, also known as decapacitation factors^5,6^.

Cysteine-rich secretory protein 1 (CRISP1) is a member of the CRISP subgroup of proteins which belongs to the CRISP, antigen 5 and Pr-1 (CAP) superfamily^7^. CRISP1 is expressed by the epithelia of the epididymis and secreted into the lumen of the epidydimal duct where it associates with the sperm surface during spermatozoa maturation^8^. CRISP1 is a multifunctional protein playing diverse roles during fertilization^9^. CRISP1 associates with the sperm surface with two different affinities during epididymal maturation^10^. Loosely bound CRISP1 is released during capacitation and is suggested to function as a decapacitation factor^11^, whereas the tightly bound CRISP remains bound after capacitation and participates in sperm-zona pellucida (ZP) interactions^10,12^. During the acrosome reaction, CRISP1 relocates to the equatorial segment of the sperm head (*i.e*. the specific surface area of sperm involved in the fusion during fertilization)^13^. Using a knockout mouse model system, CRISP1 was shown to affect sperm function without, however, leading to a significant reduction in fertility^14,15^. A double knockout of CRISP1 and CRISP4 in mice revealed that mutant males exhibit a phenotype of impaired fertility^16^. CRISP1 is also present in the cumulus cell layer surrounding the ovulated oocyte and capable of modulating sperm motility and orientation before fertilisation^14^.

**Figure 1:**
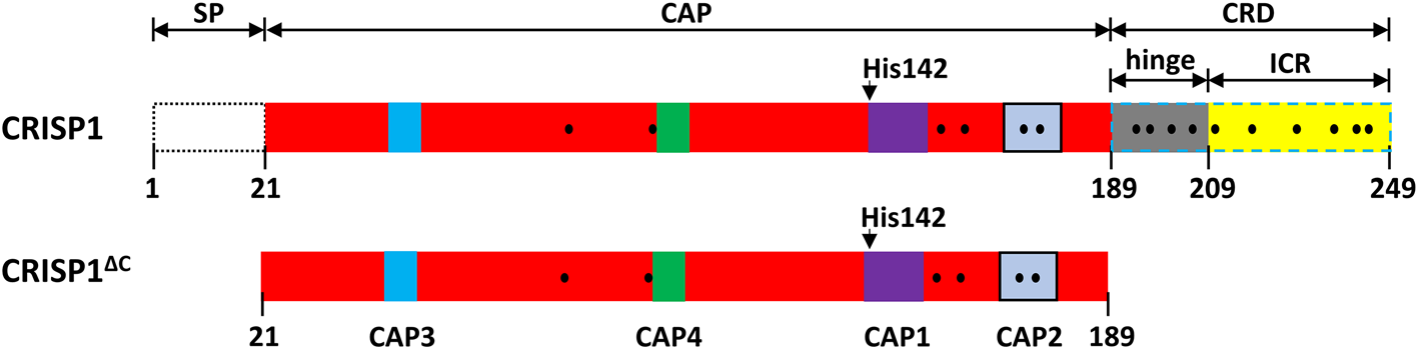
Structural characteristics of human CRISP1 and expression strategies. CRISP1 is composed of an N-terminal signal peptide (SP), a CAP domain and a C-terminal cysteine-rich domain (CRD). The CRD domain contains an ion channel regulatory (ICR) region and a hinge connecting CAP domain and ICR region. There are 16 conserved cysteine residues (black dots) in CRISP1, 6 of which are in the CAP domain and 10 are in the CRD domain. Within the CAP domain, there are four conserved CAP signature motifs (CAP1-4). His142 is a highly conserved amino acid through CAP superfamily members.

CRISP proteins are composed of an N-terminal signal peptide, a CAP domain and a C-terminal cysteine-rich domain (CRD)^7^. CRISPs are characterized by 16 cysteine residues, 10 of which are located in the CRD domain (Fig. 1 and Supplementary Figure 1). All sulfhydryl groups of these cysteine residues can be involved in intradomain disulfide bond formation^17–20^. The CRD domain contains an ion channel regulatory (ICR) region and a hinge that connects the ICR region to the CAP domain^7^.

The structurally conserved CAP domain contains four signature motifs (CAP1-4) that define the CAP superfamily^7^. Another characteristic of the CAP superfamily is the presence of two conserved histidines and glutamates on either side of a cleft across the protein surface of the CAP domain^7^. An increasing number of studies show that these two histidines form a zinc-binding site^21–25^. Although the biological function of zinc-binding is not known, we recently showed that Zn^2+^-binding to another CAP family member, GAPR-1, can trigger amyloid-like oligomerization^24^. Also several other CRISP proteins were shown to bind Zn^2+^ (*e.g*. natrin^22^ and pseudecin^23^), resulting in the formation of high-molecular-weight complexes (*e.g*. rat CRISP1^21^, mouse CRISP2 and mouse CRISP4^24^). Furthermore, rat CRISP1 zinc-dependent oligomers were shown to play a functional role in sperm epididymal maturation, suggesting that Zn^2+^-dependent oligomerization may have functional relevance^21^. In this respect it is interesting to note that zinc-dependent oligomerization and amyloid-like fibril formation of GAPR-1 is reversible^26^, providing a potential mechanism for regulation of biological activity of CAP superfamily members via the CAP domain^27,28^.

Therefore, the Zn^2+^-dependent oligomerization properties of human CRISP1 were investigated. Human CRISP1 is one of the few family members that lack one of the characteristic histidine residues that are involved in Zn^2+^ binding. In the case of human CRISP1, only His142 is conserved, whereas His86 has been replaced with glutamate (Supplementary Figure 1). This substitution provides a negative charge, potentially permitting an alternative means for Zn^2+^ coordination together with the conserved His142. Here, the question is addressed whether human CRISP1 can still bind Zn^2+^ to form oligomers that are believed to function in the reproductive process.

## Materials and methods

### Reagents

Restriction enzymes and DNA ligase were purchased from Thermo Fisher Scientific (Eindhoven, the Netherlands). pMAL-c2x vector and XL-1 Blue competent cells were from Biolab (Barendrecht, the Netherlands). Competent cells Origami 2(DE3) pLysS were from Merck (Darmstadt, Germany) and pG-KJE8/BL21 cells were from TaKaRa (Otsu, Japan). All primers were synthetized in Baseclear (Leiden, the Netherlands). ZnCl_2_, ethylenediaminetetraacetic acid (EDTA) and Isopropyl β-D-1-thiogalactopyranoside (IPTG) were obtained from Sigma-Aldrich (St. Louis, MO, USA), and bis(sulfosuccinimidyl)suberate (BS_3_) was from Pierce Biotechnology (Rockford, IL, USA).

### Plasmid construction

The cDNA of human CRISP1 (NM_001131) was obtained from OriGene (Rockville, MD, USA). cDNA of human CRISP1 was used as a template in a PCR reaction. 5′-cggaattcaaaaagaaatcagctagagacc-3′ was used as the forward primer, and 5′-gcaagctttcattttatctcagtgtcacaca-3′ and 5′-gcaagctttagcttataaggttcattctttg-3′ were used as reverse primers in the cloning of full-length CRISP1 and of the CAP domain of CRISP1 (CRISP1^ΔC^) PCR products into pMAL-c2x vector, respectively. The amplified PCR products were inserted into digested vectors according to the standard protocols. All constructs were verified by DNA sequencing (Baseclear, Leiden, the Netherlands).

### Site direct mutagenesis of pMAL-c2x-CRISP1 H142Q and pMAL-c2x- CRISP1^ΔC^ H142Q

pMAL-c2x-CRISP1 and pMAL-c2x-CRISP1^ΔC^ constructs were used as templates. pMAL-c2x-CRISP1 H142Q and pMAL-c2x-CRISP1^ΔC^ H142Q mutants were generated by site-directed mutagenesis using 5′-gatgatgacataactactgaccaatacactcagattgtttgggcc-3′ as the forward primer and 5′-ggcccaaacaatctgagtgtattggtcagtagttatgtcatcatc-3′ as the reverse primer. Mutations were verified by DNA sequencing (Baseclear, Leiden, the Netherlands).

### Expression strategies for MBP-CRISP1/MBP-CRISP1^ΔC^

The human CRISP1 constructs that were transformed into different *E. coli* strains are summarized in Supplementary Figure 2A. The *E. coli* pG-KJE8/BL21 strain possesses two chaperone systems that enhance the solubility of proteins^29–31^ and the Origami 2(DE3) pLysS strain improves disulfide bond formation in the cytoplasm of *E. coli*^32^. Initially, the human CRISP1 coding sequence was optimized according to the codon bias of *E.coli*^33^ and fused with the N-terminal His-tag. His-CRISP1 was overexpressed in the three different bacterial strains (pG-KJE8/BL21, Origami 2(DE3) pLysS and XL-1 Blue) but analysis of expression of the resulting recombinant proteins showed that under these conditions, His-CRISP1 remained mainly insoluble (Supplementary Figure 2B).

**Figure 2:**
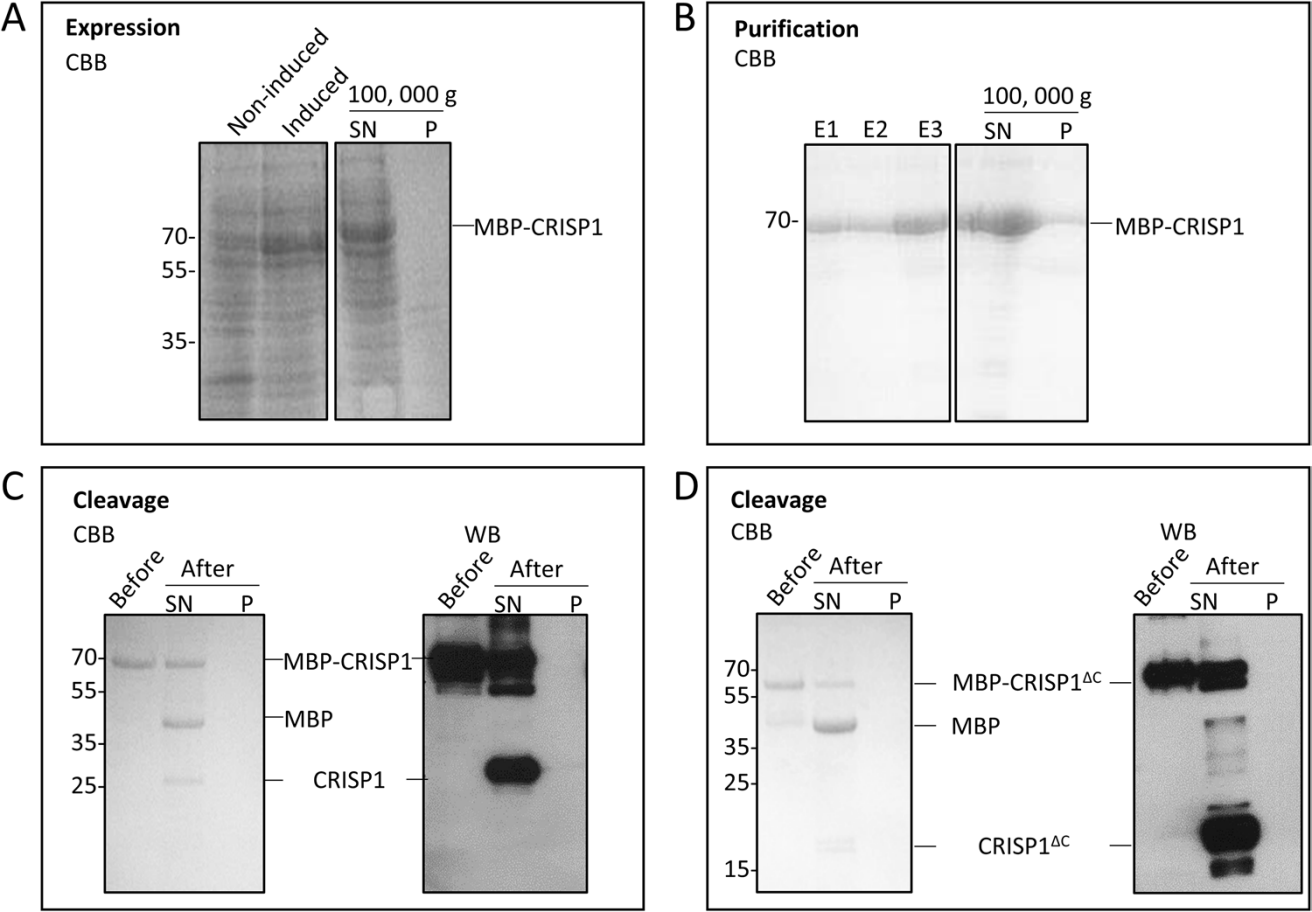
Expression and purification of CRISP1/CRISP1^ΔC^ in XL-1 Blue bacteria. (**A**) Expression of MBP-CRISP1. Samples before (Non-induced) or after induction (Induced) with IPTG were centrifuged at 100,000 g and the supernatant (SN) and pellet (P) of induced samples were analyzed by SDS-PAGE and Coomassie Brilliant blue (CBB). (**B**) Purification of MBP-CRISP1. MBP-CRISP1 was eluted with maltose (elution fractions (E1-E3)). Elution fraction E3 was centrifuged at 100,000 g and the supernatant (SN) and pellet (P) were analyzed by SDS-PAGE and Coomassie Brilliant blue. (**C**, **D**) Cleavage of MBP-CRISP1 and MBP-CRISP1^ΔC^. Before and after cleavage of MBP-CRISP1 (**C**) and MBP-CRISP1^ΔC^ (**D**) with Factor Xa, the fractions were centrifuged at 100,000 g and the supernatant (SN) and pellet (P) were analyzed by SDS-PAGE and Coomassie Brilliant blue staining or Western blotting (WB).

To improve the solubility of the target proteins, we employed solubility enhancing tags, *i.e.* maltose-binding protein (MBP) and glutathione S-transferase (GST) tags^34^. To this end, MBP or GST was fused to the N-terminus of CRISP1. Solubility of the recombinant proteins were tested. MBP-CRISP1 showed better solubility characteristics than GST-CRISP1 expressed in XL-1 Blue cells (Supplementary Figure 3). Expression of MBP-CRISP1 in the specialized bacterial strains Origami 2(DE3) or pG-KJE8/BL21 did not benefit the expression for different reasons: MBP-CRISP1 expressed in Origami 2(DE3) *E. coli* was recovered mainly in the insoluble fraction; MBP-CRISP1 expressed in pG-KJE8/BL21 *E. coli* could not be purified to homogeneity. Moreover, after MBP-cleavage, CRISP1 became highly sensitive to degradation (Supplementary Figure 4). This indicates that untagged CRISP1 expressed and isolated from pG-KJE8/BL21 *E. coli* strain was unstable. Successful expression of MBP-CRISP1/MBP- CRISP1^ΔC^ in XL-1 Blue *E.coli* is described below.

**Figure 3:**
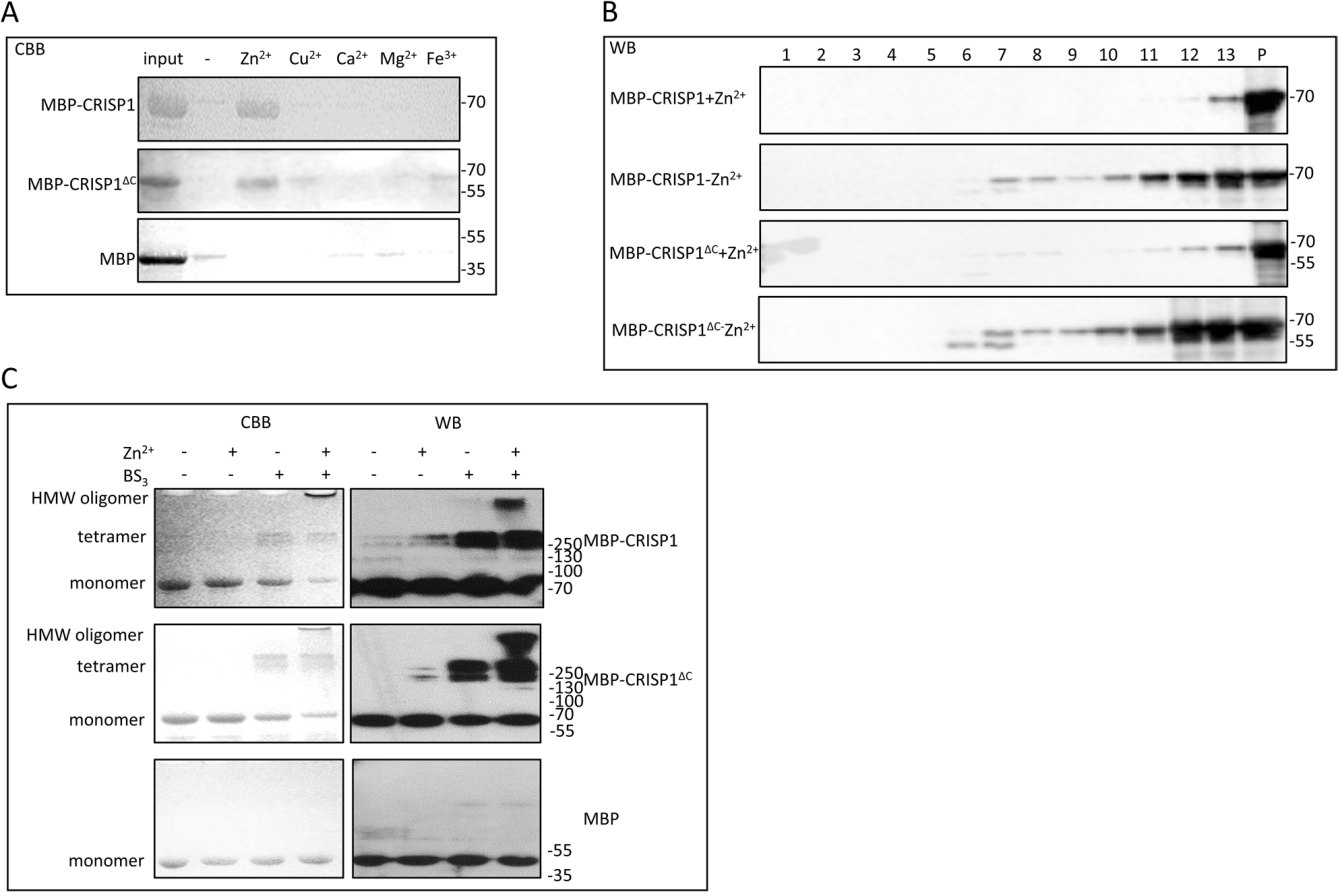
Zn^2+^-induced CRISP1 oligomerization. (**A**) 1.5 μM MBP-CRISP1/MBP-CRISP1^ΔC^/MBP was incubated in the absence or presence (1 mM) of various metal ions (Zn^2+^/Cu^2+^/Ca^2+^/Mg^2+^/Fe^3+^ as indicated) at 37 °C for 10 min. Samples were spun down at 14,000 g for 1 h at 4 °C. The pellet fraction was analyzed by SDS-PAGE gel and Coomassie Brilliant blue (CBB) staining. (**B**) 1.5 μM MBP-CRISP1/MBP-CRISP1^ΔC^ was incubated with or without 1 mM Zn^2+^ at 37 °C for 10 min. The incubations were then loaded on top of the sucrose gradient. After overnight centrifugation at 210,463 g at 4 °C, 13 fractions (fraction 1 (lowest density) to fraction 13 (highest density)) from the density sucrose gradient were collected, including the pellet (P), and precipitated by chloroform/methanol. Each fraction was analyzed by SDS-PAGE gel and Western blotting (WB). (**C**) 1.5 μM MBP-CRISP1/MBP-CRISP1^ΔC^/MBP was incubated with 1 mM Zn^2+^ at 37 °C for 10 min. Cross linker (BS_3_) was then added in a 30-fold molar excess to the incubations as indicated. Samples were analyzed by SDS-PAGE and Coomassie Brilliant blue (CBB, left panels) or Western blotting (WB, right panels).

**Figure 4:**
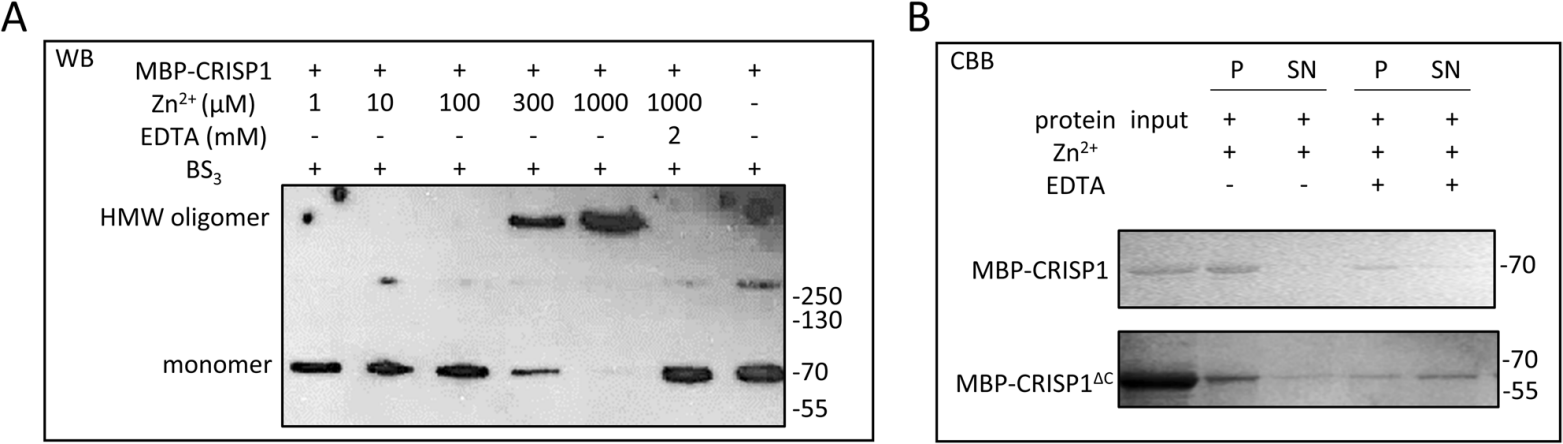
Characterization of Zn^2+^-induced CRISP1 high molecular weight complex formation. (**A**) Concentration dependency of Zn^2+^-induced human CRISP1 oligomerization. 1.5 μM MBP-CRISP1 was incubated with increasing amount of Zn^2+^ (0–1 mM) as indicated. 2 mM EDTA was added to the incubations containing 1 mM Zn^2+^. After incubation at 37 °C for 10 min, BS_3_ was added in 30-fold molar excess to the proteins. Samples were analyzed by SDS-PAGE gel and Western blotting (WB). (**B**) Reversibility of Zn^2+^-induced human CRISP1 oligomerization. 1.5 μM MBP-CRISP1/MBP-CRISP1^ΔC^ was incubated with 1 mM Zn^2+^ at 37 °C for 10 min, followed by addition of 2 mM EDTA (as indicated) and incubated at 37 °C for another 10 min. Samples without treatment by EDTA were controls. All samples were spun down at 14,000 g for 1 h at 4 °C. The pellet fraction (P) and 1/5 of the supernatant (SN) were analyzed by SDS-PAGE gel and Coomassie Brilliant blue (CBB) staining.

### Expression of MBP-CRISP1/MBP-CRISP1^ΔC^ in XL-1 Blue *E.coli* strain

pMAL-c2x-CRISP1/pMAL-c2x-CRISP1^ΔC^ was transformed into competent XL-1 Blue cells. Transformed bacteria were grown in LB media supplemented with 50 μg/ml ampicillin. The preculture was grown overnight (ON) at 37 °C and split 1/100 into fresh LB media with 50 μg/ml ampicillin. After bacteria grew to OD_600_ of 0.5, protein expression was induced with 0.5 mM IPTG and the bacteria were incubated ON at 16 °C. Bacteria were harvested by centrifugation at 4,000 g for 30 min at 4 °C.

The MBP-CRISP1/MBP-CRISP1^ΔC^ bacterial pellets were resuspended in the column buffer (50 mM Tris, 200 mM NaCl, 1 M arginine, pH 7.4, 0.6 μM aprotinin, 1 μM leupeptin-hemisulfate, 1.45 μM pepstatin and 0.5 mM phenylmethylsulfonyl fluoride (PMSF)) at a ratio of 5 ml buffer per g of bacteria pellet. The bacteria were crushed with a high-pressure homogenizer (Avestin, Mannheim, Germany) and centrifuged at 4,000 g for 30 min at 4 °C. The resultant supernatant was centrifuged again at 100,000 g at 4 °C for 30 min. Expression was analyzed by SDS-PAGE and Coomassie Brilliant blue staining.

### Purification and cleavage of MBP-fusion protein

The soluble fraction after 100,000 g centrifugation was mixed with the amylose resin (Biolab, Barendrecht, the Netherlands) and placed in a chromatography column (Biolab, Barendrecht, the Netherlands) at room temperature for 2 h. Non-specifically bound proteins were removed by washing with 10 column volumes (CVs) of washing buffer (50 mM Tris, 200 mM NaCl, pH 7.4) at 4 °C. The protein was eluted with 10 mM maltose in elution buffer (25 mM Tris, 50 mM NaCl, pH 7.4, 0.6 μM aprotinin, 1 μM leupeptin-hemisulfate, 1.45 μM pepstatin and 0.5 mM PMSF) at 4 °C. The eluate was centrifuged at 100,000 g for 30 min at 4 °C to remove aggregated proteins. The purity and solubility of isolated proteins were analyzed by SDS-PAGE and Coomassie Brilliant blue staining. To remove the MBP tag, the elute was incubated with 1% Factor Xa (Thermo Fisher Scientific, Eindhoven, the Netherlands) at room temperature overnight. The cleaved products were analyzed by SDS-PAGE and Coomassie Brilliant blue staining and/or Western blotting using rabbit polyclonal anti-CRISP1 antibody (Abcam, Cambridge, UK).

### Sedimentation analysis

Protein aggregation was analyzed by sedimentation behavior as described previously^26^. In short, MBP-CRISP1 (0.1 mg/ml)/MBP-CRISP1^ΔC^ (0.09 mg/ml)/MBP (0.06 mg/ml) was incubated in the absence or presence of 1 mM Zn^2+^, Cu^2+^, Ca^2+^, Mg^2+^ or Fe^3+^ in 25 mM Tris, 50 mM NaCl, pH 7.4, in a total volume of 100 μl reaction for 10 min at 37 °C, followed by centrifugation at 14,000 g for 1 h at 4 °C. Proteins in the pellet fraction were analyzed by SDS-PAGE and Coomassie Brilliant blue staining.

### Sucrose density gradient centrifugation

Discontinuous sucrose density gradients were prepared by gently layering sucrose densities solutions with decreasing sucrose densities (w/v) on top of one another: 0.5 ml 35%, 0.5 ml 30%, 0.8 ml 25%, 1 ml 20% and 1 ml 15% sucrose (bottom to top) in Beckman Ultra-Clear tubes (Brea, CA, USA).

1.5 μM MBP-CRISP1/MBP-CRISP1^ΔC^ was incubated in the absence or presence of 1 mM Zn^2+^ in 25 mM Tris, 50 mM NaCl, pH 7.4, in a total volume of 1 ml reaction. After incubation at 37 °C for 10 min, the reaction mixture was gently loaded on top of the gradient. The gradient was centrifuged at 210,463 g overnight at 4 °C. After centrifugation, fractions of 380 μl were gently collected from top to bottom of the gradient.

Proteins in every fraction were precipitated by chloroform/methanol. Briefly, 1.14 ml chloroform/methanol (1:2) was added to each gradient fraction sample of 380 μl. The sample was then mixed and centrifuged at 13,000 g for 1 h at 4 °C. The supernatant was removed and the protein pellet was air-dried. The samples were dissolved in Laemmli sample buffer, loaded on a 12% SDS-PAGE gel and analyzed by Western blotting using rabbit polyclonal anti-CRISP1 antibody (ab251805 from Abcam, Cambridge, UK).

### Protein crosslinking

1.5 μM MBP-CRISP1/MBP-CRISP1^ΔC^/MBP was incubated in the absence or presence of 1 mM Zn^2+^ at 37 °C in 20 mM Hepes buffer, pH 7.4, in a total volume of 40 μl. After 10 min incubation, each reaction was equally divided into two groups. BS_3_ (bis(sulfosuccinimidyl)suberate) was added in 30-fold molar excess to the protein to one group and incubated for 15 min at room temperature. 0.5 M Tris, pH 7.4 was added to quench the reaction. The other group served as control samples (absence of crosslinker). Proteins and crosslinked products were analyzed by SDS-PAGE and subsequently visualized by Coomassie Brilliant blue or Western blotting using rabbit polyclonal anti-CRISP1 antibody (Abcam, Cambridge, UK).

## Results

### Expression and purification of recombinant human CRISP1 and CRISP1^ΔC^

To investigate the oligomerization properties of human CRISP1 and the role of the CAP domain in this process, we aimed to overexpress and purify both full-length human CRISP1 and a C-terminal deletion mutant containing only the CAP domain of CRISP1 (CRISP1^ΔC^) as shown in Fig. 1.

Heterologous overexpression of CRISP proteins has been proven difficult^35–37^. Different bacterial expression systems were employed with different induction conditions and different tags (His, GST, MBP) to improve the solubility, expression levels and purification of human CRISP1 (see Supplementary Figure 2 for an overview and Materials and Methods for more details). Successful overexpression of MBP-CRISP1/CRISP1^ΔC^ was achieved in the cloning strain XL-1 Blue. The majority of overexpressed MBP-CRISP1 or MBP-CRISP1^ΔC^ remained soluble after expression in XL-1 Blue and remained stable after multiple freeze–thaw cycles. To optimize the expression and recovery of MBP-CRISP1/CRISP1^ΔC^ in XL-1 Blue cells, the experimental conditions were varied, including temperature (16–37 °C), IPTG concentration (0.5–1 mM), duration of protein induction (3–18 h), as well as the presence of various molecules known to improve protein stability (arginine, DTT, glycine). Protein expression was found to be optimal at 16 °C with IPTG (0.5 mM) induction for 18 h and addition of 1 M arginine to the homogenate. Figure 2A shows the expression and solubility characteristics of recombinant MBP-CRISP1 in XL-1 Blue cells.

The isolated MBP-CRISP1 was soluble and could be purified to apparent homogeneity by affinity-based purification using the MBP-tag (Fig. 2B). To obtain tag-free CRISP1 or CRISP1^ΔC^, the purified constructs were treated with Factor Xa. After cleavage, tag-free CRISP1/CRISP1^ΔC^ remained soluble, but the cleavage efficiency was relatively low (Fig. 2C,D). In the case of CRISP1, removal of the tag resulted in the formation of oligomers. For optimal stability and solubility during storage and freeze/thaw cycles, CRISP1 and CRISP1^ΔC^ were therefore stored as MBP-tagged fusion proteins (MBP-CRISP1 and MBP-CRISP1^ΔC^) at -80 °C. Under these optimized conditions, the yield of MBP-CRISP1 and MBP-CRISP1^ΔC^ after purification was 1.2 mg/l and 1.7 mg/l bacterial culture, respectively.

### Zn^2+^ induces human CRISP1 oligomerization by interaction with the CAP domain

Zn^2+^ interaction with the CAP domain is coordinated by two highly conserved histidine residues^21–25^. As human CRISP1 contains only one of the conserved histidines (His142, see also Fig. 1 and Supplementary Figure 1), we investigated the Zn^2+^-dependent oligomerization properties of human CRISP1. Sedimentation analysis showed that after incubation with Zn^2+^, both MBP-CRISP1 and MBP-CRISP1^ΔC^ were recovered in the pellet fraction (Fig. 3A). This sedimentation behavior was due to an effect of Zn^2+^ on the CRISP1 protein and not due to the MBP-tag as purified MBP did not sediment under these conditions. Other metal ions, including Cu^2+^, Ca^2+^, Mg^2+^ and Fe^3+^ did not have an effect on MBP-CRISP1 or MBP-CRISP1^ΔC^ sedimentation (Fig. 3A).

To confirm that human CRISP1 oligomerizes in the presence of Zn^2+^, a sucrose density gradient assay was performed in which monomers of CRISP1 are recovered in the low-density fraction whereas CRISP oligomers appear in the high-density fraction. MBP-CRISP1 or MBP-CRISP1^ΔC^ was incubated in the absence or presence of Zn^2+^ and subsequently loaded on top of the sucrose gradient (see Materials and Methods). After centrifugation, both MBP-CRISP1 and MBP-CRISP1^ΔC^ migrated from low-density to high-density sucrose fractions in the presence of Zn^2+^ (Fig. 3B), indicating the formation of oligomeric structures. Additional evidence for the Zn^2+^-dependent formation of oligomeric CRISP1 proteins was provided by a crosslink assay using an amine-to-amine crosslinker (BS_3_). Figure 3C shows that in the presence of Zn^2+^, both MBP-CRISP1 and MBP-CRISP1^ΔC^ formed high molecular weight oligomers (Fig. 3C, lanes 4 and 8, respectively), whereas MBP did not oligomerize in the presence of Zn^2+^. In the absence of crosslinker, no CRISP1 high molecular weight oligomers could be detected (Fig. 3C, lanes 2 and 6). To determine the dose-dependency of Zn^2+^ induced CRISP1 oligomerization, isolated MBP-CRISP1 was incubated with various concentrations (0.001–1 mM) of Zn^2+^ ions. CRISP1 formed high molecular weight oligomers in the presence of 0.3 mM Zn^2+^ or at higher concentrations (Fig. 4A). EDTA effectively inhibited the formation of oligomers (Fig. 4A), confirming the role of Zn^2+^ in this process. These combined results show that Zn^2+^ specifically induces human CRISP1 oligomerization, which is triggered by the zinc ion coordination to His142 of the CAP domain of human recombinant CRISP1. The MBP-tag itself is not involved in this oligomerization process.

### Zn^2+^ induced human CRISP1 oligomerization is reversible

Our previous study on GAPR-1 amyloid-like aggregation showed that removal of Zn^2+^ caused a partial disintegration of the Thioflavin T (ThT)-positive structures of GAPR-1^26^. The potential reversibility of Zn^2+^ mediated human CRISP1 and CRISP1^ΔC^ oligomerization was investigated by a sedimentation assay. Addition of EDTA to both Zn^2+^ induced MBP-CRISP1 and MBP-CRISP1^ΔC^ oligomers resulted in decreased protein recovery in the pellet fraction (Fig. 4B). This sedimentation behavior indicates that upon release of Zn^2+^, oligomers formed by both full-length and CAP domain of human CRISP1 can be (partially) dissociated again. These results indicate a reversible nature of Zn^2+^-induced human CRISP1 oligomers.

### His142 is essential for Zn^2+^ regulated human CRISP1 oligomerization

**Figure 5:**
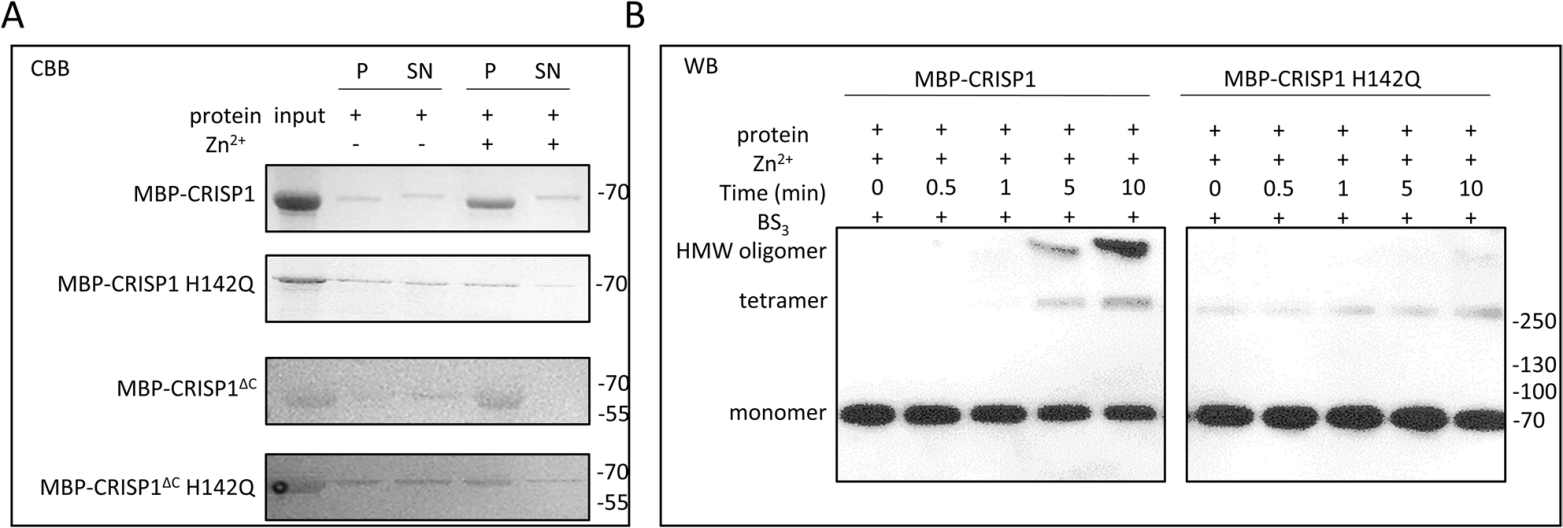
His142 is essential in Zn^2+^-induced human CRISP1 oligomerization. (**A**) 1.5 μM MBP-CRISP1/MBP-CRISP1 H142Q/MBP-CRISP1^ΔC^/MBP-CRISP1^ΔC^ H142Q was incubated with 1 mM Zn^2+^ at 37 °C for 10 min, respectively. The reactions were spun down at 14,000 g for 1 h at 4 °C. The pellet fraction (P) and 1/5 of the supernatant (SN) were analyzed by SDS-PAGE gel and Coomassie Brilliant blue (CBB). (**B**) 1.5 μM MBP-CRISP1/MBP-CRISP1 H142Q was incubated with 1 mM Zn^2+^ at 37 °C for 10 min. BS_3_ was added in 30-fold molar excess to CRISP1 and incubated for different time points (0, 0.5, 1, 5 and 10 min). Samples were analyzed by SDS-PAGE gel and Western blotting (WB).

The metal-binding site of human CRISP1 is less conserved by replacement of His for Glu at amino acid position 86. To investigate whether the metal binding-site consisting of the conserved His142 and potentially Glu86 is still involved in the Zn^2+^-dependent oligomerization properties of human CRISP1, His142 was mutated to glutamine and subsequently a sedimentation analysis was performed to analyze the effect of Zn^2+^ on the oligomerization properties of MBP-CRISP1 H142Q and MBP-CRISP1^ΔC^ H142Q. Zn^2+^-induced sedimentation of both MBP-CRISP1 H142Q and MBP-CRISP1^ΔC^ H142Q after centrifugation was significantly diminished relative to the equivalent unmutated proteins (Fig. 5A).

To confirm the absence of high-molecular-weight human CRISP1 oligomers after substitution of His142 with glutamine, MBP-CRISP1 and MBP-CRISP1 H142Q were incubated with Zn^2+^ at 37 °C for different times and subjected to the cross-link assay (Fig. 5B). Indeed, Zn^2+^ did not induce high-molecular-weight oligomers of MBP-CRISP1 H142Q, whereas MBP-CRISP1 oligomers were observed. These results confirm an essential role of His142 residue in Zn^2+^-dependent human CRISP1 oligomerization.

## Discussion

The structural complexity conferred on CRISP proteins by the high cysteine content has hampered their expression and purification with satisfactory solubility, purity, stability and yield. Attempts to express and purify mouse CRISP4 and mouse Tpx-1 from bacterial cells resulted in the formation of insoluble protein aggregates^35,36^. Human and mouse CRISP3 expression in mammalian cells resulted in low expression levels, low yields, and impure protein fractions^37^. Expression of His-tagged porcine CRISP1 has also been attempted in porcine endometrial glandular epithelial (PEGE) cells and Chinese hamster ovary cells, but the production was minimal^38^. In this study, we report the expression and purification of CRISP1 and CRISP1^ΔC^ in *E. coli* in amounts that are sufficient for biochemical studies. MBP-tagging of the CRISP1 protein, in combination with low expression levels, provided optimal conditions for obtaining soluble protein. These low expression levels were achieved with a reduced temperature during induction and by expression in XL-1 Blue bacterial cells. Decreasing the induction temperature is frequently used as one of the strategies to obtain soluble proteins. At lower temperatures, bacteria grow slowly and have a lower protein expression level, resulting in a lower frequency of protein misfolding and aggregation^39,40^. This strategy allowed us to obtain sufficient recombinant protein to demonstrate that the metal-ion dependent oligomerization properties of CAP family members is conserved in human CRISP1.

The presence of a CAP domain is a characteristic feature of CAP superfamily proteins^7^. It has been proposed that oligomerization and amyloid formation is a common functionality of the CAP domain^27^. This was recently demonstrated by using GAPR-1 as a model protein of the CAP domain^24^. Zn^2+^ binding to the metal-binding site induces GAPR-1 amyloid-like aggregation in the presence of heparin, suggesting that zinc ion-regulated oligomerization could be a common structural property of the CAP domain with potential relevance for all other CAP proteins^24^. In agreement with this, several CAP family members, including GAPR-1^24^ and CRISP subfamily members (rat CRISP1^21^, mouse CRISP2^24^, mouse CRISP4^24^, and natrin (a CRISP member present in cobra venom^22^)), form high molecular weight complexes/oligomers in the presence of Zn^2+^. Similar to GAPR-1, the proposed Zn^2+^-binding site of rat CRISP1 is formed by the two histidine residues in the CAP domain^21^. These two histidines are highly conserved throughout the CAP superfamily of proteins with the exception of only a few family members (see for example Supplementary Figure 1; human CRISP1 and mouse CRISP4). The first histidine residue in the CAP1 signature motif is conserved in all family members. The second histidine resides between the CAP3 and CAP4 signature motifs and is not entirely conserved, with replacement of histidine for glutamate (hCRISP1) or aspartate (mouse and rat CRISP4). Glutamate, aspartate and cysteine residues have been identified as protein ligands for zinc atoms^41^. Therefore, it is likely that Glu86 is involved in Zn^2+^ coordination together with His142 within the conserved metal-binding site in human CRISP1, resulting in Zn^2+^-dependent oligomerization.

For most CAP family members, Zn^2+^ is coordinated by two histidines and mutating one of the two histidines in GAPR-1 for alanine abrogated the Zn^2+^-dependent fibril formation^24^. Here we show that some variability is permitted for the histidine residue residing between the CAP3 and CAP4 signature motifs. Replacement of this histidine for glutamate (hCRISP1) still permits Zn^2+^-dependent oligomerization. However, this substitution may result in less efficient Zn^2+^ coordination. In agreement with this, we find that hCRISP1 forms high-molecular-weight oligomers at Zn^2+^ concentrations ≥ 300 µM, which is much higher than the half-maximal Zn^2+^ concentration of ~ 50 µM required for the formation of amyloid-like fibrils of GAPR-1^24^.

A reduced Zn^2+^ sensitivity of hCRISP1 could be an adaptation to the reproductive tract. Zinc is a key element for growth and development and important for the normal functioning of the reproductive system^42–45^. Apart from CRISP proteins, also other native zinc-binding high molecular weight multiprotein complex have been identified, for example from human seminal plasma^46^. The concentration of Zn^2+^ ions is increasing from proximal to distal regions of the epididymal lumen, with low zinc concentration found in the caput and corpus epididymides and high levels of Zn^2+^ demonstrated in the cauda epididymides^47,48^. High Zn^2+^ concentrations have been reported in the epididymis, with concentrations reaching 1 mM in the epididymis of prepubertal rats, and 2 mM in dog and human seminal plasma, respectively^49–51^. The Zn^2+^ concentrations used to induce CRISP1 oligomerization in this study are in the same range (0.3–1 mM) and could therefore be of potential physiological relevance. It has been reported that increasing concentrations of Zn^2+^ in the epididymis play a role in modulating the association of loosely bound rat CRISP1 to sperm surface during epididymal transit and with the presence of rat CRISP1 high-molecular-weight structures in the cauda epididymal fluids^21^. Altogether, this indicates that CRISP1 oligomerization could play a functional role in sperm epididymal maturation by increasing concentrations of Zn^2+^ in the fluid of the epididymal lumen.

Crosslinking studies of recombinant human CRISP1 showed the presence of tetramers and high molecular weight oligomers. This behavior is reminiscent of crosslinking studies with GAPR-1 showing the presence of GAPR-1 dimers and high molecular weight oligomers^52,53^. In addition, GAPR-1 migrates as a diffuse band at approximately the dimer-tetramer molecular weight range as shown by Blue Native Gel electrophoresis^26^. The fact that CRISP1 forms tetramers rather than dimers may be related to the high cysteine content of this protein. The presence of tetrameric CRISP1 is in agreement with a previously published model describing the presence of relatively stable native multimers that shift to monomers prior to the formation of oligomers^26^. In this model, specific and redox-dependent dimeric arrangements of GAPR-1 may affect distinct amyloid-like oligomerization pathways and it is tempting to speculate that the redox-dependent disulfide bond formation may affect the oligomerization process of CRISP1 is similar ways. We previously showed that zinc-mediated GAPR-1 aggregation was (partially) reversed upon addition of EDTA^26^. In addition, the high-molecular-weight structures of rat CRISP1 present in the cauda epididymal fluid partially disintegrated by EDTA-mediated Zn^2+^ chelation^21^. Our present study revealed that EDTA also caused the disintegration of oligomers of either full-length CRISP1 or C-terminally truncated human CRISP1 (representing the CAP domain). These results suggest that differential Zn^2+^ concentrations within the reproductive organs may allow spatiotemporal oligomerisation of CRISP proteins by a dynamic and reversible process. Functional protein aggregates play diverse roles in reproduction and fertilization, *e.g*. in hormone peptides storage^54,55^, oocyte dormancy^56,57^, acrosome reaction^58^, zona pellucida (ZP) formation^59^, epididymal sperm maturation^60^, and in sperm-ZP recognition and fusion^61^. In contrast to pathological protein aggregation, many functional protein aggregates are reversibly regulated^54,55,58,62–64^. Amyloid aggregates in the acrosomal matrix (AM) are described to be essential for the stability of the AM core which is crucial for sperm-ZP penetration. During acrosome reaction, the disintegration of amyloid aggregates is central for the AM dispersion^58^. The loosely bound population of CRISP1 is released during sperm capacitation which is concomitant with a decrease in Zn^2+^ levels^10^. Our combined results may provide potential novel insights into the molecular regulation of human CRISP1 function in the reproductive physiology of sperm. Metal ion-regulated oligomerization could be a common structural property of CAP domain-containing proteins^24^. We recently showed that the CAP1 and CAP2 signature motifs contain amyloidogenic propensities, suggesting that amyloid-like aggregation is a common and evolutionary conserved property of this protein family^27,65^. Metal-ion binding could provide a general switch, allowing (reversible) protein oligomerization of CAP family members, permitting execution of their specific biological function. In the case of CRISP1 proteins, we speculate that low Zn^2+^ levels may play a role in the initial steps of capacitation by allowing the disassembly of CRISP1 oligomers from membranes during sperm activation.

## Supplementary Information

Supplementary information.

## Abbreviations

CAP: Cysteine-rich secretory proteins, antigen 5, and pathogenesis-related-1
CRISPs: Cysteine-rich secretory proteins
GAPR-1: Golgi-associated plant pathogenesis related protein-1
MBP: Maltose binding protein
ZP: Zona pellucida
CRD: Cysteine rich domain
ICR: Ion channel regulatory
ThT: Thioflavin T
EDTA: Ethylenediaminetetraacetic acid
IPTG: Isopropyl β-D-1-thiogalactopyranoside
BS_3_: Bis(sulfosuccinimidyl)suberate
ON: Overnight
PMSF: Phenylmethylsulfonyl fluoride
