# Supplementary material for: The less conserved metal-binding site in human CRISP1 remains sensitive to zinc ions to permit protein oligomerization
Source: Sci Rep. 2021 Mar 9;11:5498. doi: 10.1038/s41598-021-84926-y (PMC7943821; doi:10.1038/s41598-021-84926-y)
Supplement: Supplementary file 1 — Supplementary information. [file 41598_2021_84926_MOESM1_ESM.pdf]

The less conserved metal-binding site in human CRISP1 remains sensitive to zinc ions to permit protein oligomerization

Jie Sheng, Bart M. Gadella, Nick K. Olrichs, Dora V. Kaloyanova, J. Bernd Helms*

Department of Biomolecular Health Sciences, Faculty of Veterinary Medicine, Utrecht University, Utrecht, the Netherlands

* To whom correspondence should be addressed: J.B.H. (J.B.Helms@uu.nl)

Keywords: CRISP1, purification, oligomerization, zinc, CAP domain

Supplementary figure legends

Supplementary Figure 1. Sequence alignment of GAPR-1 and CRISP proteins from mammals and snake venom. The predicted amyloidogenic regions, obtained from amyloid prediction tool FoldAmyloid (Garbuzynskiy, S. O., Lobanov, M. Y. & Galzitskaya, O. V. FoldAmyloid: A method of prediction of amyloidogenic regions from protein sequence. *Bioinformatics* **26**, 326–332 (2009)) are highlighted in green. CAP signature motifs and 16 conserved cysteine residues (*) in CRISP proteins are indicated below the sequences. Two conserved histidine residues and two conserved glutamates involved in the metal binding site of CAP superfamily proteins are highlighted with red boxes. The semi-conserved cysteine in GAPR-1 (Cys63) is indicated with red arrow.

Supplementary Figure 2. Expression of His-CRISP1 in pG-KJE8/BL21, Origami 2 (DE3) pLysS, and XL-1 Blue. (A) Different tags and bacterial strains were used to obtain CRISP1/CRISP1$^{\Delta C}$. His, GST or MBP tag was fused to the N-terminus of CRISP1/CRISP1$^{\Delta C}$ (without SP). Protein expression was performed in pG-KJE8/BL21, Origami 2(DE3) pLysS and XL-1 Blue strain, respectively. (B) Expression of MBP-CRISP1 in pG-KJE8/BL21. Samples before (Non-induced) or after induction (Induced) with IPTG were centrifuged at 100,000 *g* and the supernatant (SN) and pellet (P) of induced samples were analyzed by SDS-PAGE and Coomassie Brilliant blue (CBB). (C) Expression of MBP-CRISP1 in Origami 2 (DE3) pLysS. After induction (Induced) with IPTG, the samples were centrifuged at 100,000 *g* and the supernatant (SN) and pellet (P) were analyzed by SDS-PAGE and Coomassie Brilliant blue (CBB). (D) Expression of MBP-CRISP1 in XL-1 Blue. After induction (Induced) with IPTG, the samples were centrifuged at 100,000 *g* and the supernatant (SN) and pellet (P) were analyzed by SDS-PAGE and Western blotting (WB).

Supplementary Figure 3. Solubility of MBP-CRISP1 and GST-CRISP1 expressed in XL-1 Blue. Purified MBP-CRISP1 and GST-CRISP1 were centrifuged at 100,000 *g* after incubation at room temperature for 90 min. Supernatant (SN) and pellet (P) were analyzed by SDS-PAGE and Coomassie Brilliant blue (CBB).

Supplementary Figure 4. Expression characteristics of MBP-CRISP1 in specialized bacterial strains and MBP cleveage from CRISP1. (A) Expression of MBP-CRISP1 in Origami 2 (DE3) pLysS. Samples before (Non-induced) or after induction (Induced) with IPTG were centrifuged at 100,000 *g* and the supernatant (SN) and pellet (P) of induced

samples were analyzed by SDS-PAGE and Coomassie Brilliant blue (CBB). (B) Purification and solubility (left panel), and cleavage (right panel) of MBP-CRISP1 expressed in pG-KJE8/BL21. Purified MBP-CRISP1 (Elution) was centrifuged at 100,000 g and the supernatant (SN) and pellet (P) were analyzed by SDS-PAGE and Coomassie Brilliant blue (CBB, left panel). Samples before and after MBP-CRISP1 cleavage (see Materials and Methods for details) were analyzed by SDS-PAGE and Western blotting (WB, right panel).

Supplementary Figure 5. Uncropped Coomassie blue-stained SDS-PAGE gels for Figure 2A-D and uncropped Western blots for Figure 2C and D. Dashed boxes indicate cropped areas.

Supplementary Figure 6. Uncropped Coomassie blue-stained SDS-PAGE gels for Figure 3A and C and uncropped Western blots for Figure 3B and C. Dashed boxes indicate cropped areas.

Supplementary Figure 7. Uncropped Western blots for Figure 4A and uncropped Coomassie blue-stained SDS-PAGE gels for Figure 4B. Dashed boxes indicate cropped areas.

Supplementary Figure 8. Uncropped Coomassie blue-stained SDS-PAGE gels for Figure 5A and C and uncropped Western blots for Figure 5B. Dashed boxes indicate cropped areas.

Supplementary Figure 9. Uncropped Coomassie blue-stained SDS-PAGE gels for Suppl. Fig. 2A and B and uncropped Western blots for Suppl. Fig. 2C. Dashed boxes indicate cropped areas.

Supplementary Figure 10. Uncropped Coomassie blue-stained SDS-PAGE gels for Suppl. Fig. 3. Dashed boxes indicate cropped areas.

Supplementary Figure 11. Uncropped Coomassie blue-stained SDS-PAGE gels for Suppl. Fig. 4A and B and uncropped Western blots for Suppl. Fig. 4B. Dashed boxes indicate cropped areas.

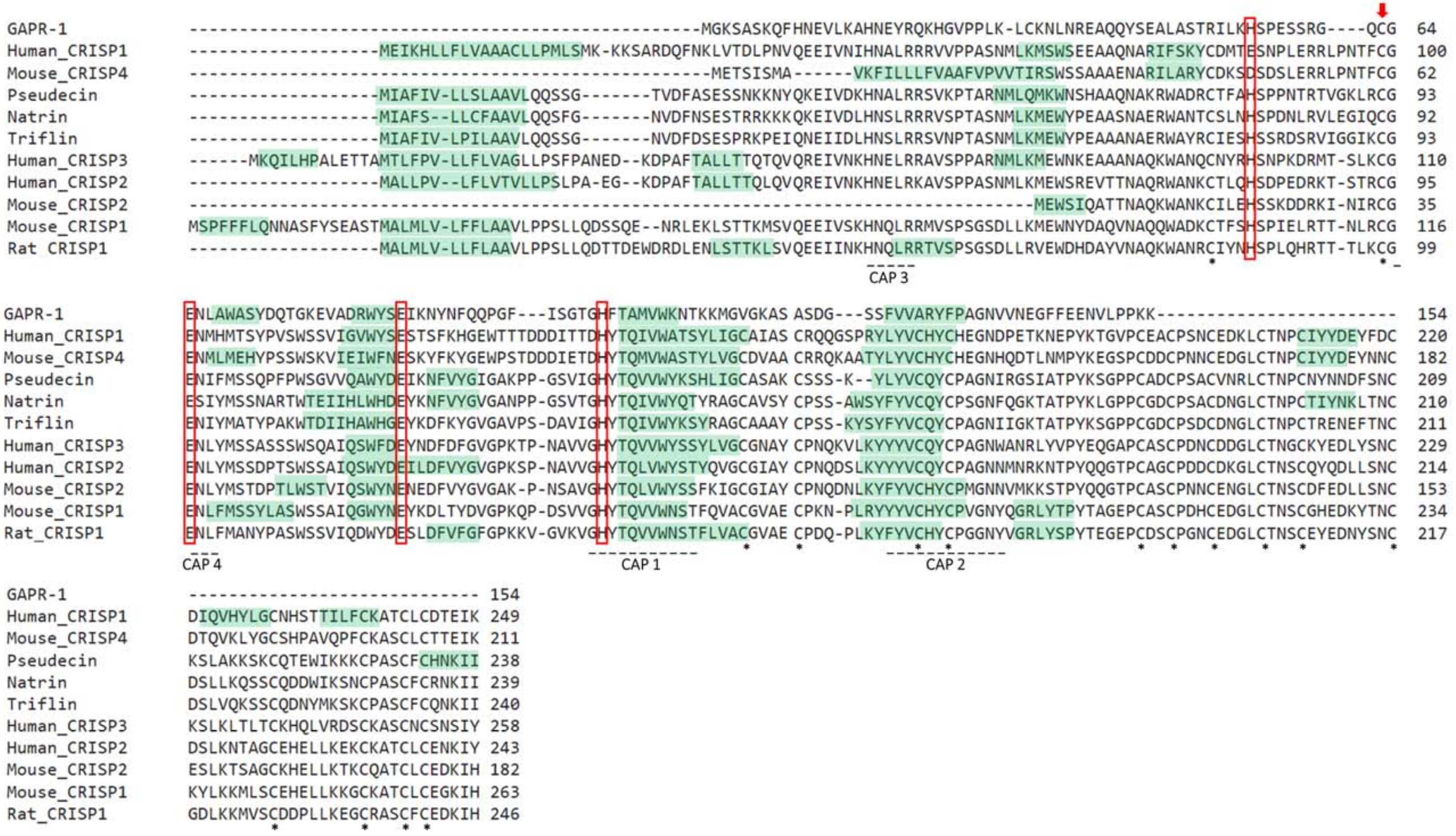

Suppl. Figure 1 Sheng et al.

A

Tag	Position	Host
His	N-terminal	pG-KJE8/BL21, Origami 2(DE3) pLysS, XL-1 Blue
GST	N-terminal	pG-KJE8/BL21, Origami 2(DE3) pLysS, XL-1 Blue
MBP	N-terminal	pG-KJE8/BL21, Origami 2(DE3) pLysS, XL-1 Blue

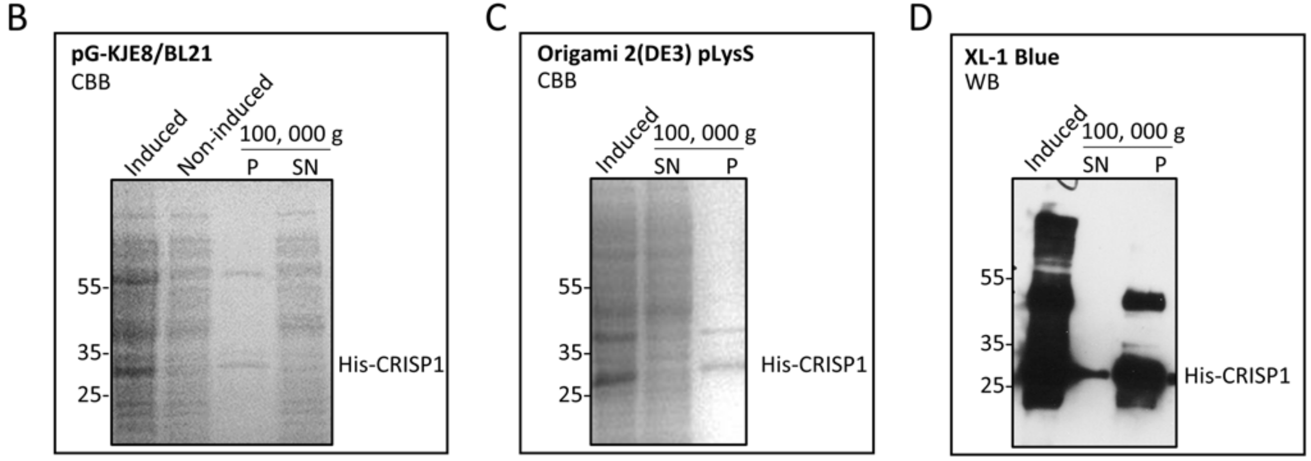

Suppl. Figure 2 Sheng et al.

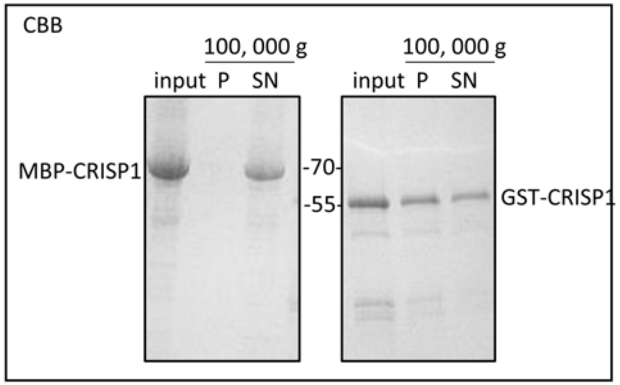

15

Suppl. Figure 3 Sheng et al.

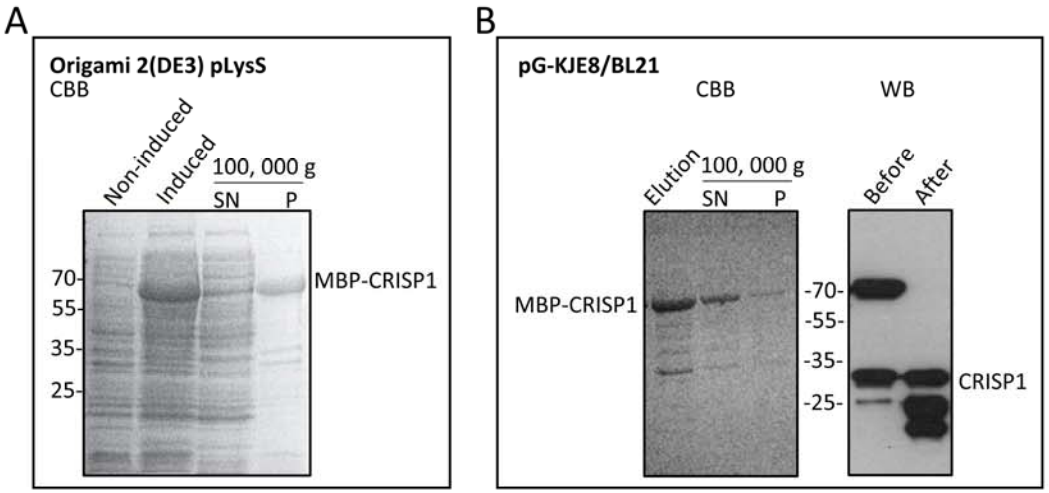

Suppl. Figure 4 Sheng et al.

Figure 2A

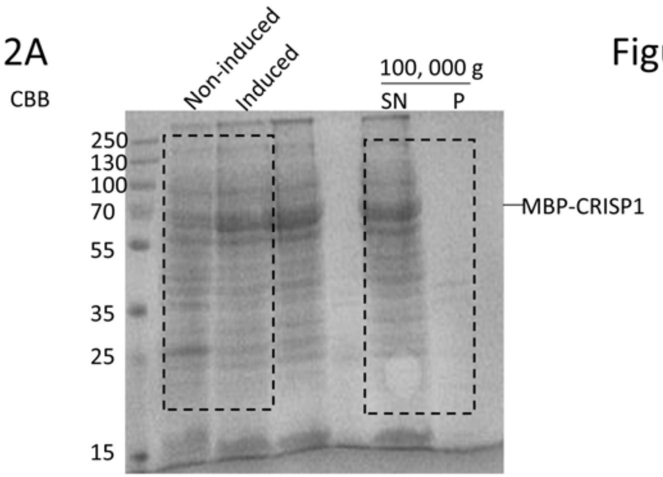

Figure 2B

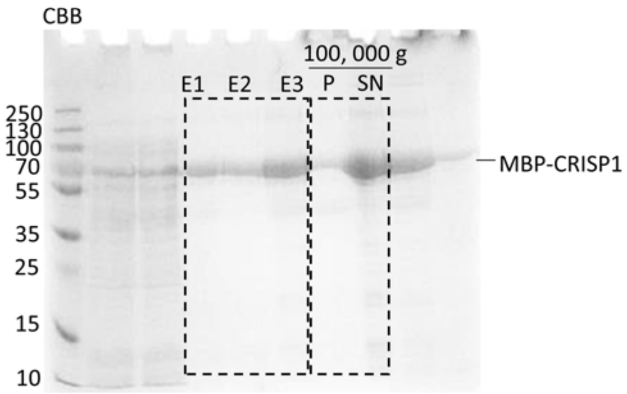

Figure 2C and D

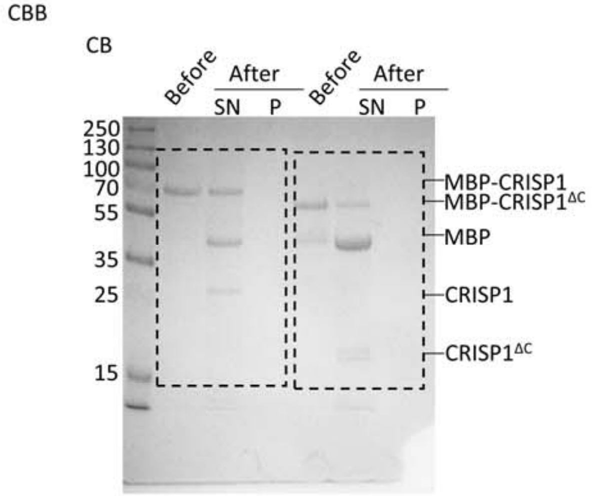

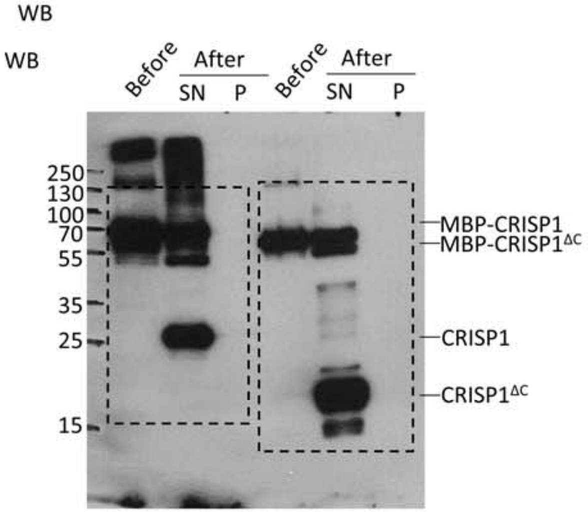

Suppl. Figure 5 Sheng et al.

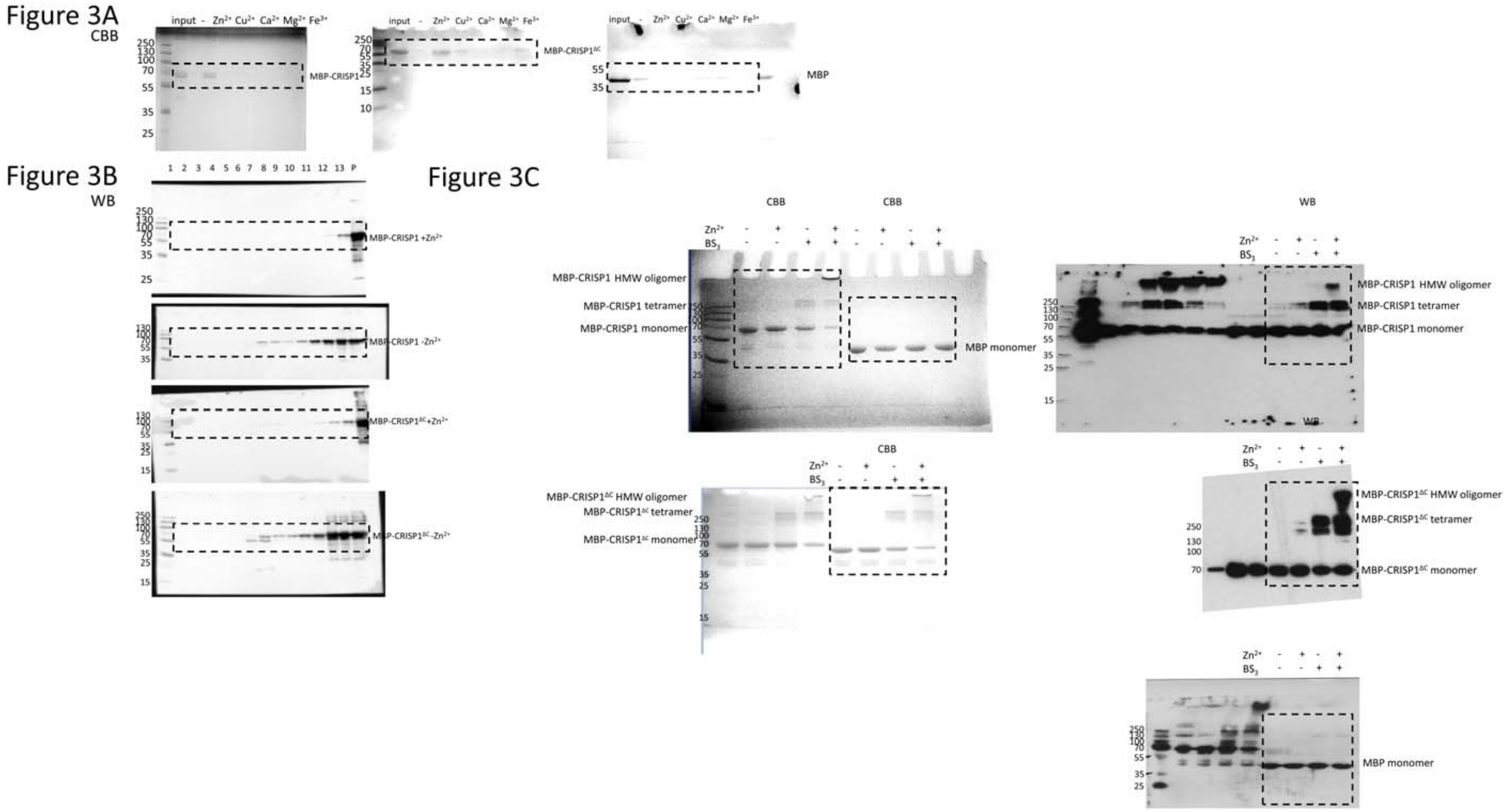

Suppl. Figure 6 Sheng et al.

Figure 4A

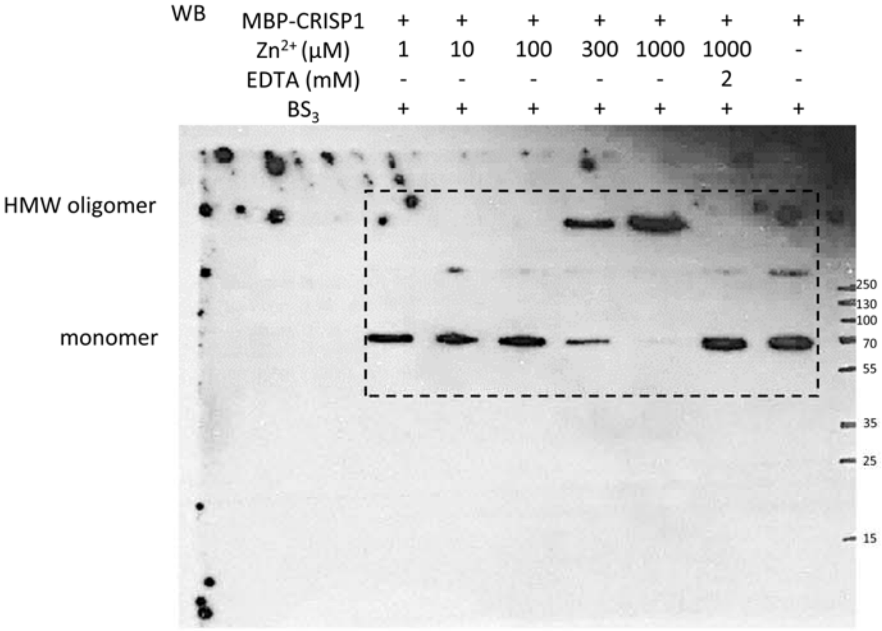

Figure 4B

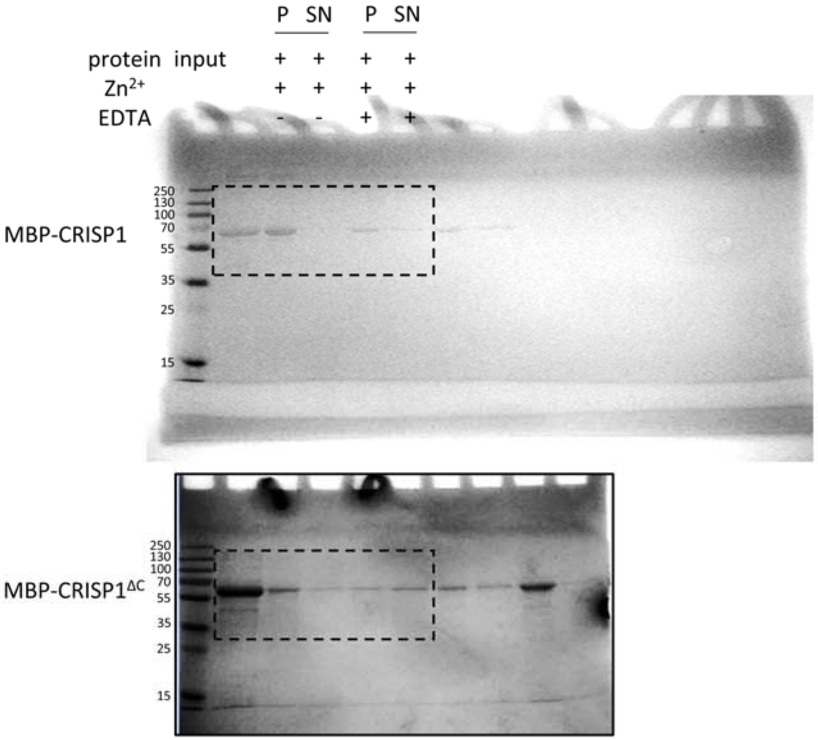

Suppl. Figure 7 Sheng et al.

Figure 5A
CBB

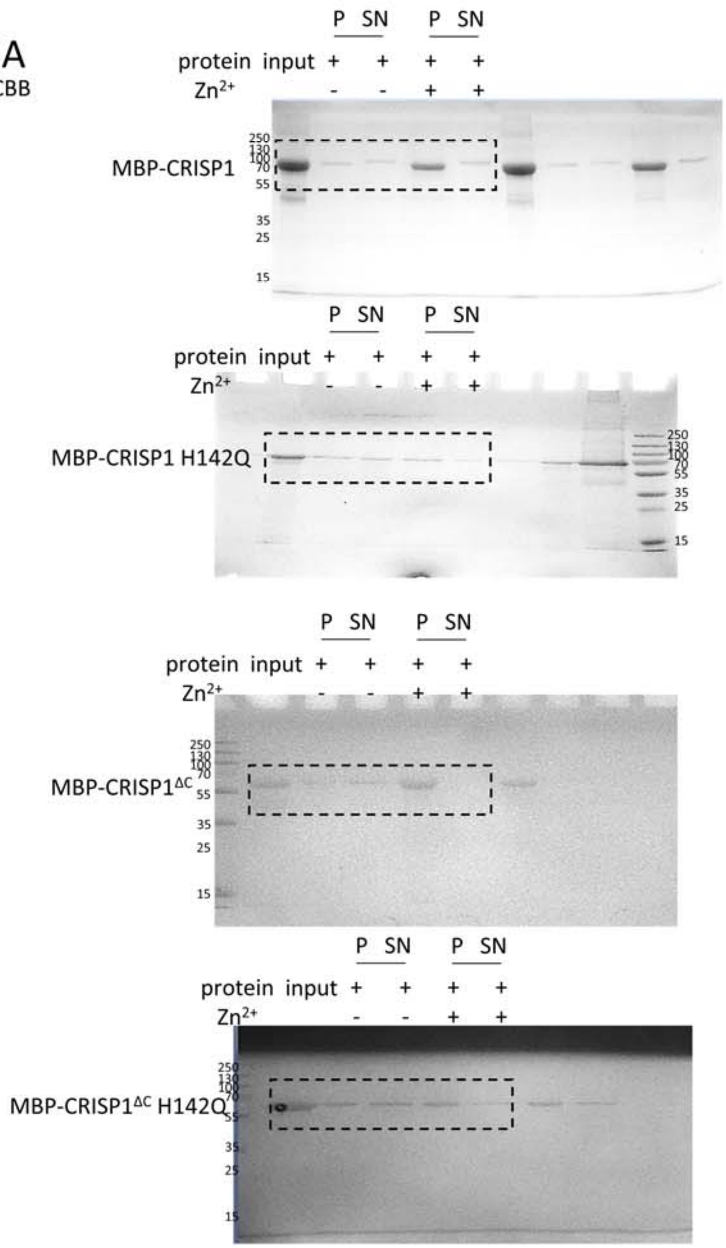

Figure 5B
WB

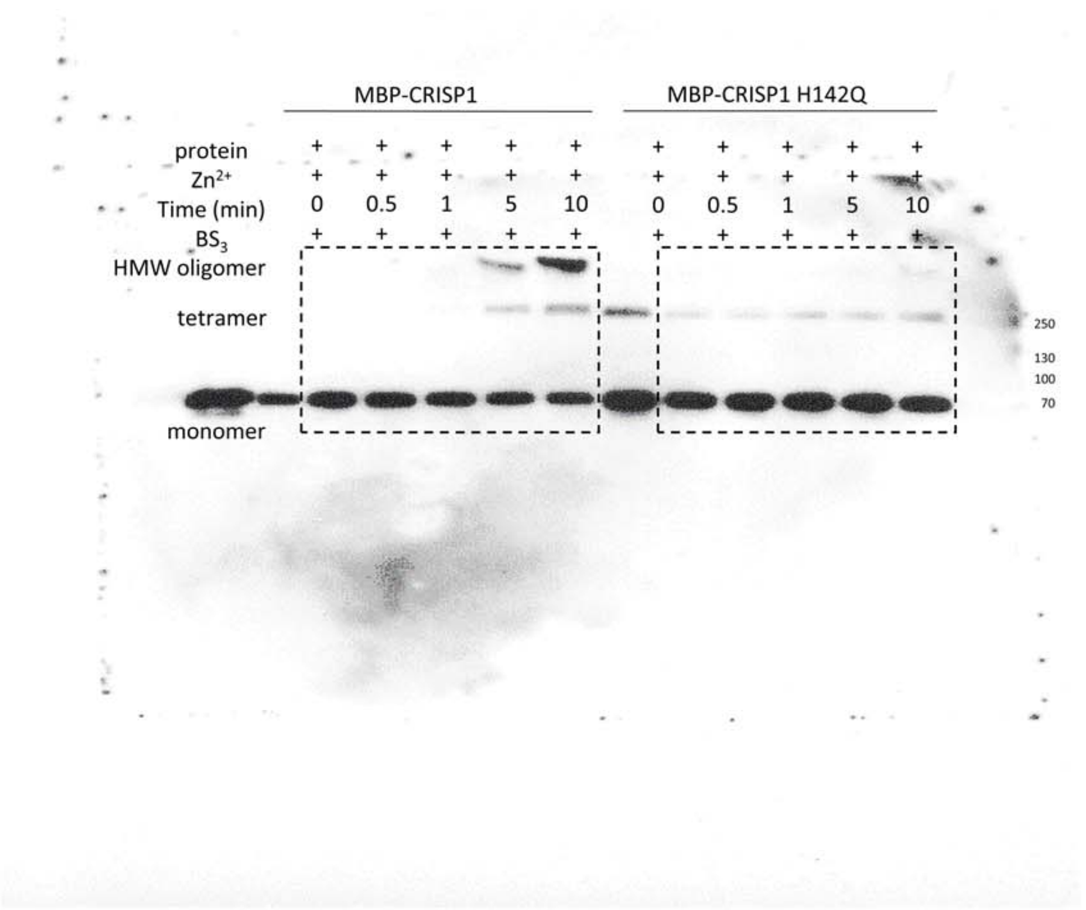

Suppl. Figure 8 Sheng et al.

Supplementary Figure 2A

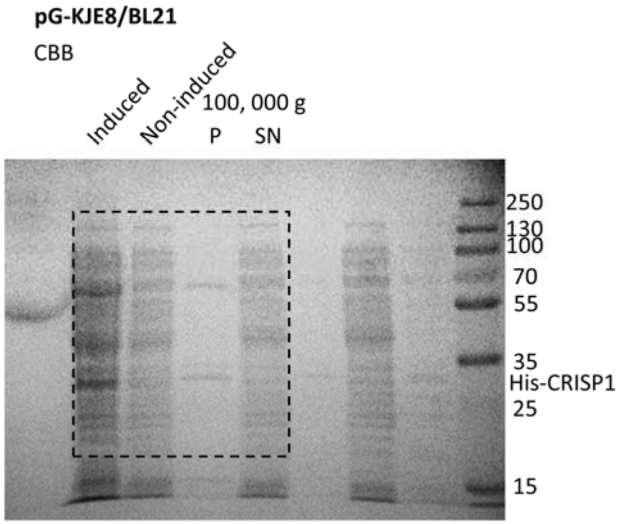

Supplementary Figure 2B

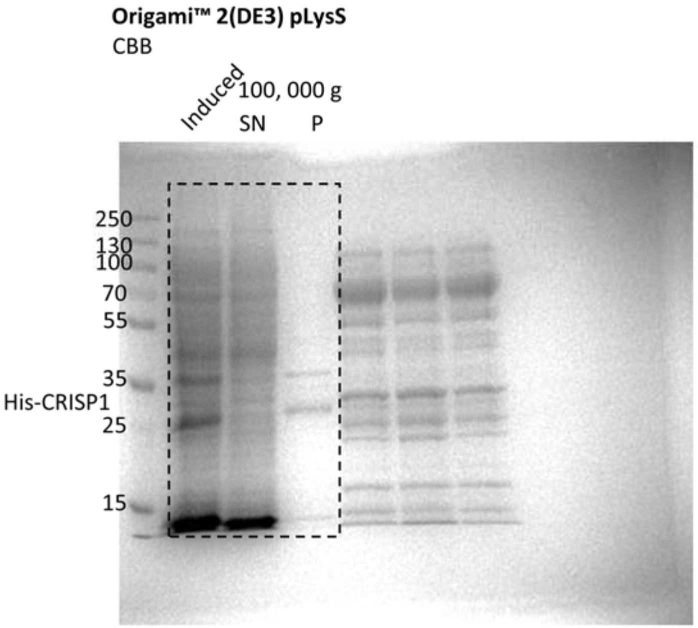

Supplementary Figure 2C

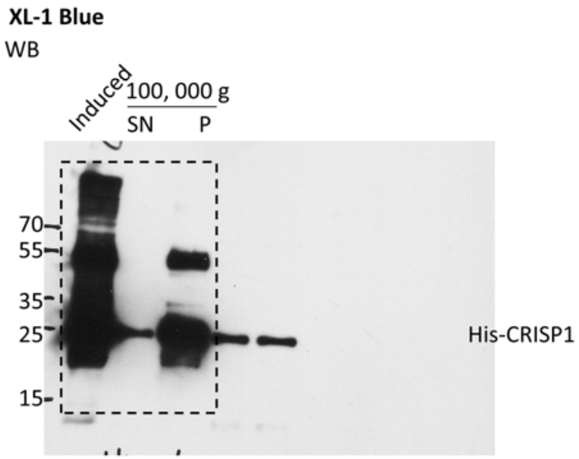

Suppl. Figure 9 Sheng et al.

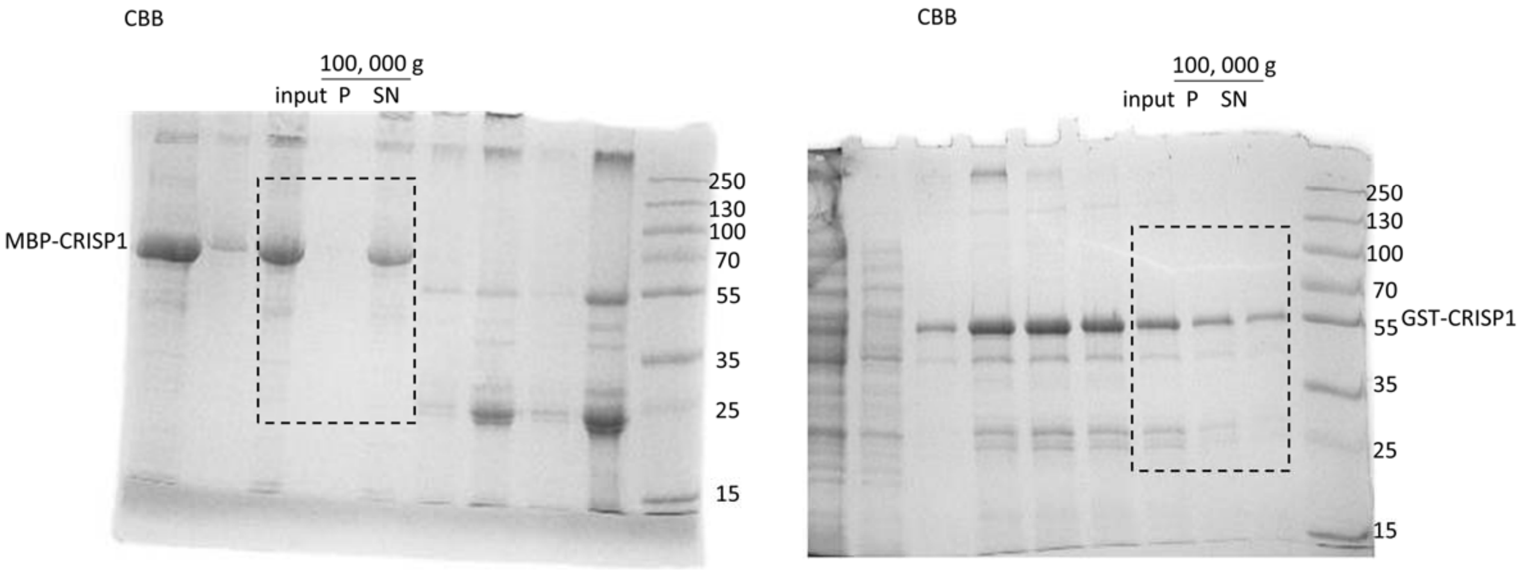

Suppl. Figure 10 Sheng et al.

Supplementary Figure 4A

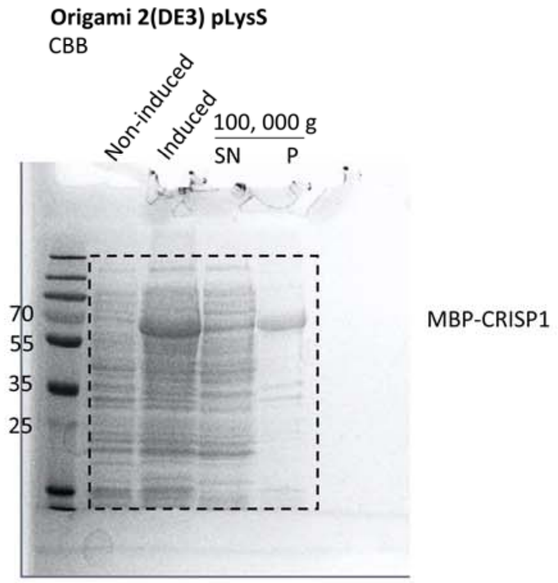

Supplementary Figure 4B

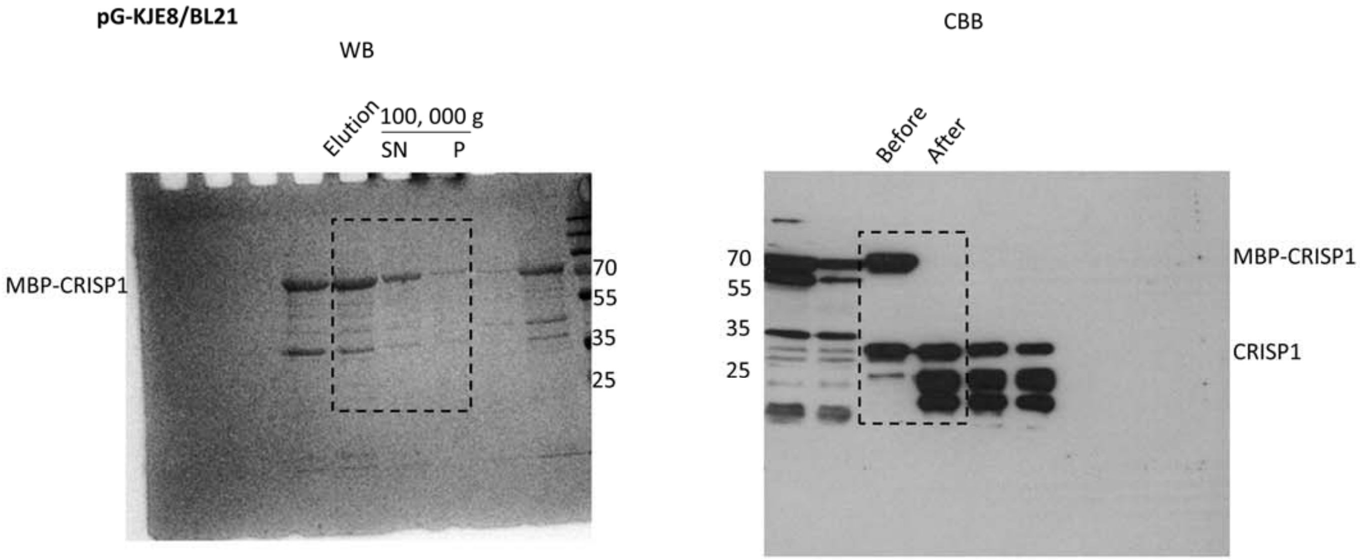

Suppl. Figure 11 Sheng et al.
